# Emergency Department-Initiated Hospice and Palliative Care Consultation Among Older Adults: Protocol for a Systematic Review and Meta-Analysis

**DOI:** 10.2196/75346

**Published:** 2026-06-30

**Authors:** Satheesh Gunaga, Kei Ouchi, Shan W Liu, Dimitri Lin, Alison Hay, Katherine Selman, Daniel Markwalter, Justin Brooten, Alex Ginsburg, Sarah Pajka, Eric Isaacs, Dennis Smythe, Kirby Swan, Naomi George, Joshua Davis, Erica Westlake, Murtaza Akhter, Naomi Rebollo Lee, Rita Manfredi, Paul Bain, Fabrice Mowbray

**Affiliations:** 1 Department of Emergency Medicine, Henry Ford Hospital, Wyandotte, MI, United States; 2 College of Osteopathic Medicine, Michigan State University, East Lansing, MI, United States; 3 Department of Emergency Medicine, Brigham and Women's Hospital, Boston, MA, United States; 4 Department of Emergency Medicine, Massachusetts General Hospital, Boston, MA, United States; 5 Department of Emergency Medicine, Harvard Medical School, Boston, MA, United States; 6 Department of Emergency Medicine, Wexford General Hospital, Wexford, Ireland; 7 Department of Emergency Medicine, Cooper Hospital University Medical Center, Camden, NJ, United States; 8 Department of Emergency Medicine, University of North Carolina at Chapel Hill, Chapel Hill, NC, United States; 9 Department of Emergency Medicine, Wake Forest University, Winston-Salem, NC, United States; 10 Department of Emergency Medicine, Mayo Clinic, Rochester, MN, United States; 11 Department of Emergency Medicine, University of Washington, Seattle, WA, United States; 12 Department of Emergency Medicine, University of California, San Francisco, San Francisco, CA, United States; 13 Department of Emergency Medicine, University of New Mexico, Albuquerque, NM, United States; 14 Department of Emergency Medicine, Penn State Milton S. Hershey Medical Center, Hershey, PA, United States; 15 Department of Emergency Medicine, Emory University Hospital, Atlanta, GA, United States; 16 Department of Emergency Medicine, Maricopa Medical Center, Phoenix, AZ, United States; 17 Department of Emergency Medicine, Columbia University, New York City, NY, United States; 18 Department of Emergency Medicine, George Washington University, Washington, DC, United States; 19 Countway Library, Harvard Medical School, Boston, MA, United States; 20 College of Nursing, Michigan State University, 1355 Bogue St, East Lansing, MI, 48824, United States, 1 2263466525

**Keywords:** palliative, hospice, emergency department, geriatrics, PRISMA, Preferred Reporting Items for Systematic Reviews and Meta-Analyses

## Abstract

**Background:**

Emergency departments (EDs) play a critical role in caring for the aging population, particularly those nearing the end of life. Despite advances in integrating palliative care resources in the ED, targeted research on the impact of hospice and palliative care (HPC) consultations for older adults in ED settings remains limited. This systematic review protocol assesses the effects of ED-initiated HPC consultations on health outcomes and the quality of care for older adults.

**Objective:**

The objective of this systematic review is to synthesize the available evidence on the effectiveness of ED-initiated HPC consultations on the provision of advance care planning among older adults (≥60 years). Our review will focus on assessing several secondary outcomes, including mortality, hospital admissions, length of stay, repeat health service use, costs, and satisfaction levels among patients, caregivers, and clinicians.

**Methods:**

Following the PRISMA-P (Preferred Reporting Items for Systematic Reviews and Meta-Analyses Protocols) reporting guidelines, our protocol outlines a comprehensive review of published studies. Systematic searches will be conducted in databases such as Medline, EMBASE, PubMed, Cochrane Trials database, and Web of Science from inception to present. Studies will be selected if they involve randomized, quasi-randomized, or observational designs examining the effectiveness of HPC interventions in the ED for older adults. Title, abstract and full text screening, risk of bias evaluation, and grading of the evidence will be completed independently and in duplicate by a group of emergency medicine and palliative care researchers. If feasible, a meta-analysis will be conducted using a random-effects model to evaluate the outlined outcomes

**Results:**

We will report descriptive statistics to describe the body of literature and we will pool absolute risk differences along with corresponding 95% CIs. We will also report on study risk of bias and certainty of the evidence.

**Conclusions:**

Despite the rapid growth of emergency medicine and HPC literature, a focused systematic review on the geriatric ED population remains absent. Our work not only fills a vital gap in the literature related to ED-initiated HCP consultations for older adults, but it sets the stage for significant future advancements in the care of older adults in the ED.

## Introduction

Between one third and half of all older adults visit the Emergency Department (ED) in their final month of life, highlighting the critical role of emergency care in identifying and addressing the specialized end-of-life needs of this population [1-4]. EDs are pivotal in the evolving landscape of health care, particularly in addressing the complex needs of an aging population [5,6]. The number of individuals aged 60 and older is expected to increase substantially on a global level over the coming years. Emergency clinicians and health systems are increasingly confronted with significant challenges in managing the complexity of this population, particularly where specialized care, like palliative care, is warranted [7,8]. The World Health Organization describes palliative care as an approach that improves quality of life for patients and families facing life threatening illness through the prevention and relief of suffering using early identification and management of physical, psychosocial, and spiritual concerns. Palliative care is also appropriate at any age and at any stage of a serious illness, and it can be provided together with curative or disease directed treatments [9]. 9 This dual role of providing acute disease–targeted care as well as facilitating access to palliative resources underscores the critical need to integrate effective palliative care strategies within the ED to improve outcomes and enhance quality of life for older adults [6].

Recent literature on the integration of hospice and palliative care (HPC) into emergency medicine has expanded exponentially over the last three decades, as evidenced by a surge in both the breadth and depth of research [10,11]. Significant advancements were catalyzed by the American College of Emergency Physicians' 2014 Choosing Wisely Campaign and the Geriatric ED Guidelines, both emphasizing the need for proactive screening and referral for specialized palliative care for older patients who may benefit [12,13]. This momentum was augmented by the 2021 United States Best Practice Guidelines, which further advocated for early ED palliative screening and HPC consultations in those with unmet needs, spurring a range of innovative studies, including prospective clinical trials [14-17]. Despite this rapid expansion, the scope and quality of evidence specifically tailored to palliative care for older adults in the ED still demand thorough examination.

In our protocol, we report on our planned efforts to describe and synthesize the currently available peer-reviewed literature evaluating the impact of ED-initiated HPC consults on patients aged 60 years and older. We hypothesize that eligible older adults who receive HPC consults in the ED have better health outcomes and quality of care, when compared to those receiving no consultation.

## Methods

We used the Preferred Reporting Items for Systematic Reviews and Meta-Analyses Protocols (PRISMA-P) to guide the reporting of our protocol (Checklist 1) [17]. The PRISMA statement will guide the reporting of our final review to ensure transparency, and we will provide a completed and detailed checklist with the publication [18]. Our review protocol was registered with the International Prospective Register of Systematic Reviews (PROSPERO), with the following registration number: CRD42024566869. We anticipate completing: record screening by January 1st, 2026, data extraction by March 2026, and dissemination of results by June 2026.

### Research Question

In older adults (≥60 y) with serious illness who present to the ED, is ED-initiated hospice and/or palliative care consultation associated with the provision of advance care planning, when compared to those who do not receive consultation? Secondary outcome of interest include:

- Mortality (ED, in-patient, post discharge 1 and 3 mo)
- Inpatient length of stay
- Hospital admission
- Repeat health service use (hospital admission and ED visitation)
- Costs (patient and health system)
- Satisfaction (patient, caregivers, clinician)

### Data Sources and Search Strategy

We consulted an academic-affiliated librarian (PAB) for the systematic literature search. We will identify studies that have examined the role of HPC consults in the ED by searching the electronic databases MEDLINE (Ovid), Embase (Elsevier), Web of Science Core Collection (Clarivate), and the Cochrane Central Register of Controlled Trials (Wiley). The searches include terms for end-of-life conversations or consultations and emergency services (Multimedia Appendix 1). Controlled vocabulary terms (MeSH, Emtree) are included when available; no date or language limit is applied. We will conduct citation tracking on all eligible studies to highlight any articles potentially missed by our search strategy.

### Study Selection and Screening

We plan to include randomized and quasi-randomized clinical trials that enrolled older ED patients (>60) with serious illness presenting for care [19]. We used the age of 60, mindful that (i) it is more inclusive that the commonly used cut-off of 65 years, and (ii) this is the definition used by the World Health Organization. We describe serious illness using the Lancet Commission framework, which defines serious illness broadly as conditions associated with serious health-related suffering that require palliative care. For ED application, serious illness includes advanced or progressive conditions with significant symptom burden, functional decline, or elevated risk of mortality that commonly prompt ED visits, such as advanced cancer, heart failure, chronic obstructive pulmonary disease, dementia, kidney disease, frailty, and severe multimorbidity [19-21]. Further mindful that this is still a relatively new body of literature, we also plan to include observational study designs, specifically purposed to evaluate HPC consultation in the ED. We plan to exclude study designs that lack a comparison group (eg, case series, case report ) Finally, we plan to exclude conference proceedings and abstracts because of limited methodological description, hindering the risk of bias (RoB) assessment. Studies will not be excluded based on language, sample size, or time of publication.

**Figure 1.**
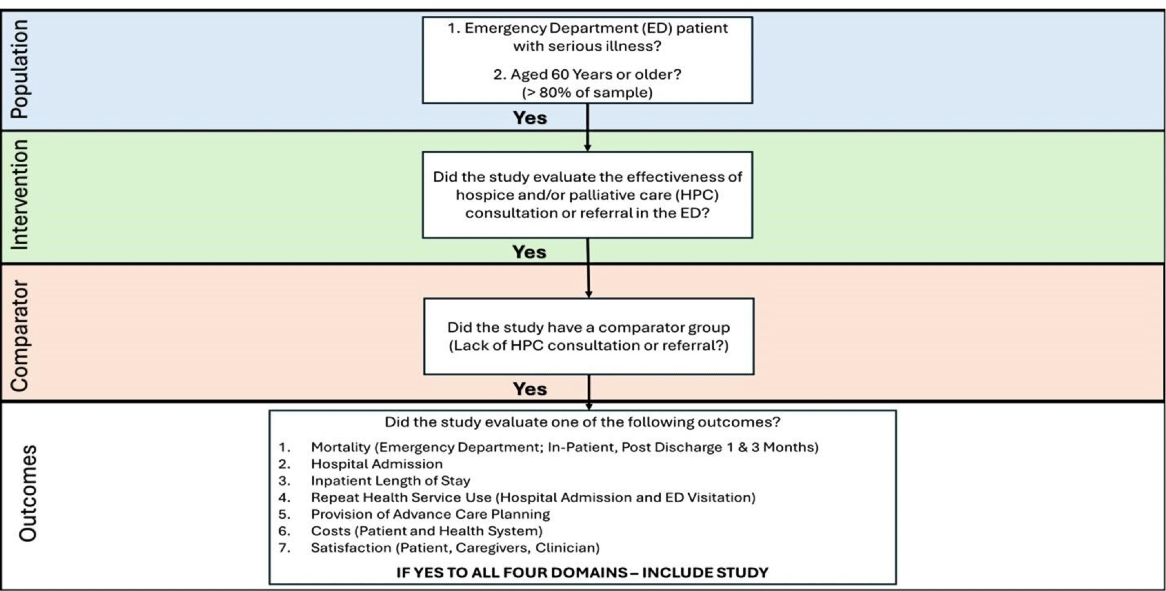
Systematic review eligibility decision tree. ED: Emergency Department; HPC: Hospice and Palliative Care.

We will export titles and abstracts into Covidence software (elbourne, Australia), where duplicates will be removed. We will conduct title, abstract, and full-text screening independently and in duplicate by two trained reviewers at minimum. A standardized decision-tree was created to guide title and abstract screening (Figure 1). Any disagreement between reviewers regarding study inclusion following title, abstract, or full-text review will be resolved through a third study reviewer and/or discussion. Inter-rater agreement on title, abstract, and full-text screening will be reported using Cohen κ statistics, respectively.

### Data Extraction

Two reviewers will extract data independently and in duplicate. A standardized and pilot-tested data collection form will be created to ensure consistency of extraction. The following data from eligible studies will be extracted: author(s), year of publication, study design, single versus multisite, country of study, inclusion and exclusion criteria, recruitment time frame (mo), follow up length, total sample size, the proportion of adults ≥60 years of age, definition and timing of outcomes, number of events, baseline demographics (eg, age, sex, frailty status/score, triage acuity), unit of change for continuous outcomes, classification for categorical outcomes, and the unadjusted and adjusted point estimates of risk with confidence intervals. All extracted data values will be rounded to two decimal places, and we will import this data into Microsoft Excel.

### Data Synthesis and Analysis

Data will be synthesized using R software (version 4.0; R Foundation for Statistical Computing) and the ‘*meta*’ package [22]. We plan on generating point estimates and their respective 95% CIs using absolute risk difference as the primary effect size, and relative measures as secondary effect sizes. Results will be pooled according to the outcome of interest, assuming the definition and timing of measurement are congruent. We plan to synthesize and report both univariable and multivariable estimates. If baseline risk estimates are not reported and conversion or data retrieval from the study materials is not possible, we will attempt to contact the study authors. Our contact process involves emailing the corresponding, first, and last authors using email addresses provided in the publication or obtained through institutional websites. If no response is received within two weeks, a follow-up email will be sent. Should contact the authors fail after one month, we will proceed with subgroup analyses based on the format of the effect estimate. We will compare studies that provide information on baseline risk, where we can appropriately convert odds ratio, risk ratio, and hazard ratio, to studies where conversion is not possible or appropriate. We will conduct a sensitivity analysis to determine the influence that data imputation has on pooled estimates. A random-effects model will be used for all statistical pooling, mindful that models of care and patient populations are likely to vary between regions [23]. Adjusted effect sizes will be converted to absolute risks using methods proposed by Foroutan and colleagues [24]. Where meta-analysis is not possible, we will report outcomes narratively.

### Risk of Bias Within Studies

We will examine the risk of bias within each study, individually and in duplicate. We will leverage the Cochrane Risk of Bias 2.0 assessment for clinical trials. Specially, the RoB will be determined by examining six domains: the randomization process, deviations from intended interventions, missing outcome data, outcome measurement, and selection of the reported result. For observational methods, we will defer to the ROBINS-I bias assessment instrument and evaluate the following domains: confounding, participant selection, intervention classification, deviations from intended intervention, missing data, outcome measurement, and reported outcomes. We will use the individual domains, rated as low or high risk of bias, to inform each study’s overall risk of bias. A flag of high risk of bias across any single domain, regardless of study design, will be classified as high risk of bias.

### Sources of Heterogeneity

Statistical heterogeneity will be assessed through the visual inspection of forest plots, examining the consistency among point estimates and overlap among the associated confidence intervals, and the *χ*^2^ test for homogeneity. The inconsistency index (*I*^2^) measure will only be used if most studies include <500 patients, mindful that prior work has demonstrated a lack of variance in the *I*^2^ measure among studies with large sample sizes [25]. Clinical and methodological heterogeneity will be examined to identify factors that may modify the association between HPC consultation and the outcomes of interest. Effect modifiers of interest are RoB (high vs low), study design (interventional vs observational), and geriatric population (mixed vs full).

### Certainty of Estimates

Certainty of our estimates will be determined using the Grading of Recommendations, Assessment, Development, and Evaluation (GRADE) approach. Per GRADE recommendations, confidence will be rated as either high, moderate, low, or very low. For meta-analyses with more than ten studies, funnel plots will be produced to examine the distribution of positive and negative findings allowing for the detection of publication bias. An individual assessment of confidence will be given for each outcome. The congruency of pooled estimates between our sensitivity analysis (including imputed non-significant studies evaluating the predictor of interest) and our primary model (not including the imputed non-significant predictor) will be reported and taken into consideration when discussing our confidence in the estimates.

## Results

We intend to report a pooled absolute risk difference as our primary effect size and corresponding 95% CIs for all outcomes where pooling is appropriate. We also intend to report synthesized findings primarily as absolute risk difference; we will also report relative measures (eg, RR, OR, etc). We will prioritize the reporting of advance care planning as our primary outcome for synthesis. For outcomes where we are unable to statistically pool results, we will provide a narrative synthesis. We will provide descriptive statistics regarding the body of literature and study characteristics (age, country, etc). We also intend to create a ‘summary of findings’ table where we will provide GRADE estimates across each outcome. Our initial search strategy resulted in 1454 articles for title and abstract screening, after removing 8 duplicate articles.

## Discussion

### Principal Findings

We are setting out to conduct a systematic review and meta-analysis, evaluating the association between ED-initiated referral for HPC and the provision of advance care planning downstream. We are also interested in evaluating the mortality, hospitalization, in-patient length of stay for those admitted, repeat health service use, costs, and satisfaction with the model of care. We hypothesized that ED-initiated referral would have a positive effect on the outcomes listed (eg, increase in provision of advance care planning, decrease in mortality, etc) We intend to disseminate our findings through presentations at academic conferences, and through the creation of a peer-reviewed publication.

Despite the expanding corpus of emergency medicine and HPC literature, a focused systematic review on the geriatric population remains conspicuously absent [10,11]. Over the past four years, numerous systematic reviews have explored various facets of EM HPC, with eight well executed systematic reviews dedicated to this domain [26-32]. However, none have specifically targeted the older adult demographic within the ED setting. This gap underscores the critical need for our systematic review protocol, which is uniquely positioned as the first to address older adult HPC consultations in the ED. The importance of this systematic review is notable, as it seeks to impact clinical outcomes, guide policy decisions, and shape future research. This ensures that emerging evidence is effectively used to improve patient care in emergency settings. By focusing on this underrepresented group, the Academy of Geriatric Emergency Medicine and the Geriatric ED Guidelines 2.0 Working Group anticipate that our findings will be instrumental in guiding the forthcoming revisions of their ED HPC guidelines, using GRADE methodology for the development of future geriatric ED clinical practice guidelines [33-35].

### Strengths and Limitations

The strengths of our protocol are manifold. It includes a well-defined Population, Intervention, Comparison, Outcome (PICO) question, the collaboration of an interdisciplinary team comprising national and international leaders in emergency medicine and HPC, and the potential to establish new standards in emergency care for older adults. Additional strengths include a comprehensive review strategy and the use of advanced analytical techniques to ensure robust synthesis of the available data. Nonetheless, the geriatric focus of our review might limit the selection of studies, potentially affecting the breadth of evidence available. Moreover, the existing literature might not yet be sufficiently developed to answer our PICO question with the desired level of reliability. Despite these limitations, our systematic review protocol still offers substantial value, supporting needs assessments by the Geriatric Evaluation & Advanced Research (GEAR) teams and contributing to the foundational knowledge required to enhance geriatric care in emergency settings [30,34-37].

### Conclusion

Our systematic review is novel in its evaluation of ED-initiated HPC consultation from the ED among a panel of patient-important outcomes among older ED patients. We hypothesize that ED-initiated HPC consultation would improve rates of advance care planning, decrease mortality, decrease hospital admission, decrease hospital length of stay, decrease costs, and improve satisfaction for patients and clinicians. The final systematic review will be utilized to develop the Geriatric ED Guidelines 2.0 recommendations related to HPC care in the ED for older adults.

## Supplementary material

10.2196/75346 Multimedia Appendix 1 Text search for article.

10.2196/75346 Checklist 1 PRISMA-P checklist.

## Abbreviations

ED: Emergency Department
GEAR: Geriatric Evaluation & Advanced Research
GEAR: Geriatric Evaluation & Advanced Research
GRADE: Grading of Recommendations, Assessment, Development, and Evaluation
HPC: Hospice and Palliative Care
PICO: Population, Intervention, Comparison, Outcome
PICO: Population, Intervention, Comparison, Outcome
PRISMA-P: Preferred Reporting Items for Systematic Reviews and Meta-Analyses Protocols
PROSPERO: Prospective Register of Systematic Reviews
RoB: Risk of Bias
