# Supplementary material for: Emergency Department-Initiated Hospice and Palliative Care Consultation Among Older Adults: Protocol for a Systematic Review and Meta-Analysis
Source: JMIR Res Protoc. 2026 Jun 30;15:e75346. doi: 10.2196/75346 (PMC13318083; doi:10.2196/75346)
Supplement: Multimedia Appendix 1 [file resprot-v15-e75346-s001.docx]

**S2 File. Electronic Database Searches.**

# MEDLINE (Ovid)

Ovid MEDLINE(R) and Epub Ahead of Print, In-Process, In-Data-Review & Other Non-Indexed Citations, Daily and Versions

1. exp emergency service, hospital/ or exp emergency medicine/ or exp emergency nursing/ or emergency treatment/ or (((emergency or casualty) adj (room? or department? or service? or unit? or ward? or facilit*)) or emergicent*).ab,kf,kw,ti.
2. (((palliative or end-of-life or eol or hospice or care goal? or "goals of care" or serious*-ill* or critical*ill* or terminal* ill*) adj3 (consult* or conversation? or discussion? or care bundle? or pathway? or transition* or transfer* or early intervention)) or goals-of-care).ab,ti,kf,kw.
3. advance directives/ or advance? directive?.ab,kf,kw,ti.
4. 1 and (2 or 3)

# Embase (1974-; Elsevier)

1. 'emergency ward'/de/mj OR 'hospital emergency service'/de/mj OR 'emergency medicine'/de/mj OR

'emergency treatment'/exp/mj OR 'emergency nursing'/de/mj OR (((emergency OR casualty) NEXT/1 (room$ OR department$ OR service$ OR unit$ OR ward$ OR facilit*)) OR emergicent*):ab,kw,ti

1. (((palliative OR 'end-of-life' OR eol OR hospice OR 'care goal$' OR 'goals of care' OR 'serious-ill*' OR 'seriously ill' OR 'critical-ill*' OR 'critically ill' OR 'terminal ill*' OR 'terminal ill*') NEAR/3 (consult* OR conversation$ OR discussion$ OR 'care bundle$' OR pathway$ OR transition* OR transfer* OR 'early intervention')) OR 'goals-of-care' OR (advance NEXT/1 directive$)):ab,kw,ti
2. #1 AND #2

# Web of Science Core Collection (Clarivate)

Exact search=”on”

1. TI=(((emergency OR casualty) NEAR/0 (room$ OR department$ OR service$ OR unit$ OR ward$ OR facilit*)) OR emergicent*) OR AB=(((emergency OR casualty) NEAR/0 (room$ OR department$ OR service$ OR unit$ OR ward$ OR facilit*)) OR emergicent*) OR AK=(((emergency OR casualty) NEAR/0 (room$ OR department$ OR service$ OR unit$ OR ward$ OR facilit*)) OR emergicent*)
2. TI=(((palliative OR "end-of-life" OR eol OR hospice OR "care goal$" OR "goals of care" OR "seriousill*" OR "seriously ill" OR "critical-ill*" OR "critically ill" OR "terminal ill*" OR "terminal ill*") NEAR/2 (consult* OR conversation$ OR discussion$ OR "care bundle$" OR pathway$ OR transition* OR transfer* OR "early intervention")) OR "goals-of-care" OR (advance NEAR/0 directive$)) OR AB=(((palliative OR "end-of-life" OR eol OR hospice OR "care goal$" OR "goals of care" OR "serious-ill*" OR "seriously ill" OR "critical-ill*"

OR "critically ill" OR "terminal ill*" OR "terminal ill*") NEAR/2 (consult* OR conversation$ OR discussion$

OR "care bundle$" OR pathway$ OR transition* OR transfer* OR "early intervention")) OR "goals-of-care"

OR (advance NEAR/0 directive$)) OR AK=(((palliative OR "end-of-life" OR eol OR hospice OR "care goal$"

OR "goals of care" OR "serious-ill*" OR "seriously ill" OR "critical-ill*" OR "critically ill" OR "terminal ill*" OR "terminal ill*") NEAR/2 (consult* OR conversation$ OR discussion$ OR "care bundle$" OR pathway$ OR transition* OR transfer* OR "early intervention")) OR "goals-of-care" OR (advance NEAR/0 directive$))

1. #1 AND #2

# Cochrance Central Register of Controlled Trials (Wiley)

Word variants=off

1. (((emergency OR casualty) NEXT/1 (room? OR department? OR service? OR unit? OR ward? OR facilit*)) OR emergicent*):ti,ab,kw
2. (((palliative OR "end-of-life" OR eol OR hospice OR "care goal" OR "care goals" OR "goals of care" OR "seriously ill" OR "serious illness" OR "serious illnesses" OR "critically ill" OR "critical illness" OR "critical illnesses" OR "terminally ill" OR "terminal illness" OR "terminal illnesses") NEAR/3 (consult* OR conversation? OR discussion? OR "care bundle" OR "care bundles" OR pathway? OR transition* OR transfer* OR "early intervention")) OR "goals-of-care" OR (advance NEXT/1 directive?)):ti,ab,kw
3. #1 AND #2

# CINAHL Complete (EBSCO)

1. MH ("Emergency Service" OR "Emergency Care" OR "Emergency Nursing+") OR TI (((emergency

OR casualty) N1 (room# OR department# OR service# OR unit# OR ward# OR facilit*)) OR emergicent*) OR AB (((emergency OR casualty) N1 (room# OR department# OR service# OR unit# OR ward# OR facilit*)) OR emergicent*)

1. TI (((palliative OR "end-of-life" OR eol OR hospice OR "care goal" OR "care goals" OR "goals of care" OR "seriously ill" OR "serious illness" OR "serious illnesses" OR "critically ill" OR "critical illness" OR "critical illnesses" OR "terminally ill" OR "terminal illness" OR "terminal illnesses") N3 (consult* OR conversation# OR discussion# OR "care bundle" OR "care bundles" OR pathway# OR transition* OR transfer* OR "early intervention")) OR "goals-of-care" OR (advance W1 directive#)) OR AB (((palliative OR

"end-of-life" OR eol OR hospice OR "care goal" OR "care goals" OR "goals of care" OR "seriously ill" OR

"serious illness" OR "serious illnesses" OR "critically ill" OR "critical illness" OR "critical illnesses" OR

"terminally ill" OR "terminal illness" OR "terminal illnesses") N3 (consult* OR conversation# OR discussion#

OR "care bundle" OR "care bundles" OR pathway# OR transition* OR transfer* OR "early intervention")) OR "goals-of-care" OR (advance W1 directive#))

1. S1 AND S2
